# Cloacal Gram-Negative Microbiota in Free-Living Grass Snake *Natrix natrix* from Poland

**DOI:** 10.1007/s00284-020-02021-3

**Published:** 2020-05-19

**Authors:** Aleksandra Pawlak, Katarzyna Morka, Stanisław Bury, Zuzanna Antoniewicz, Anna Wzorek, Gabriela Cieniuch, Agnieszka Korzeniowska-Kowal, Mariusz Cichoń, Gabriela Bugla-Płoskońska

**Affiliations:** 1 Department of Microbiology, Faculty of Biological Sciences, Institute of Genetics and Microbiology, University of Wrocław, Stanisława Przybyszewskiego 63-77, 51-148 Wrocław, Poland; 2 Institute of Environmental Sciences, Jagiellonian University, Gronostajowa 7, 30-387 Kraków, Poland; 3 Department of Immunology of Infectious Diseases, Hirszfeld Institute of Immunology and Experimental Therapy, Polish Academy of Sciences, Weigla 12, 53-114 Wrocław, Poland

## Abstract

Reptiles appear to be an important vector for Gram-negative pathogens, therefore, they are epidemiologically relevant. However, the composition of reptilian microbiota has been poorly recognized so far. The majority of studies concern exotic reptiles as asymptomatic carriers of *Salmonella* serovars. Studies of other intestinal bacteria of reptiles are rare. Only recently, the microbiota of free-living European reptiles have been investigated, however, on the basis of small samples, mainly in protected areas. Here, we aim to investigate cloacal Gram-negative microbiota of free-living *Natrix natrix.* Snakes (*N* = 45) used in the study were collected in Kraków (Poland) and its vicinity. Nineteen species of Gram-negative bacteria were isolated. The most common species were: *Aeromonas hydrophila*, *Morganella morganii*, *Proteus vulgaris, Salmonella* spp*.* The bacteria prevalent in *N. natrix* cloacal swabs are likely to represent the natural intestinal Gram-negative microbiota of the examined snakes. Importantly, the identified bacteria are pathogenic to humans, which clearly highlights the epidemiological potential of free-living *N. natrix*. The risk of infection is high for immunocompromised humans, children (under 5 years old), elderly persons, and pregnant women. Our study provides the largest dataset on intestinal Gram-negative microbiota of wild snakes. The presence of multiple human pathogens determined by us calls for the necessity of further studies on reptile-transmitted bacteria in anthropogenic environments.

**Electronic supplementary material:**

The online version of this article (10.1007/s00284-020-02021-3) contains supplementary material, which is available to authorized users.

## Introduction

The intestinal microbiota of reptiles has not yet been sufficiently investigated. The majority of the studies focus on exotic reptiles as asymptomatic carriers of *Salmonella* serovars [1–5]. Studies of other bacterial species from intestinal microbiota of reptiles are rare and usually conducted on exotic reptiles [6]. So far, only a few studies on free-living European reptiles have been reported [7, 8]. Studies of the microbiota of free-living reptiles in Poland are very limited and most of them also concern *Salmonella* spp. as a microbiota component [9–12]. Most of exotic reptiles are asymptomatic carriers of *Salmonella* serovars. However, transmission of these pathogens to humans can lead to a serious infection in risk groups, such as children under 5, pregnant women or immunocompromised patients. Two terms have been used to name such infection: RAS (reptile associated salmonellosis) and REPAS (reptile-exotic pet associated salmonellosis) [13, 14]. Term REPAS has been proposed to emphasize the role of exotic species of reptiles, as the studies on European reptiles show that although they may also be carriers of *Salmonella* serovars, this is not that frequent as in the case of exotic reptiles [14]. However, more research is needed to properly determine the differences between the intestinal microbiota of exotic and domestic European species of reptiles. A number of publications have shown that RAS/REPAS could lead to serious infections, which can end up with septicemia, miscarriage or death [3, 4, 15–19]. That is why European reptiles should be screened. Reptiles can also be carriers of other bacteria which are pathogenic for people, such as: *Morganella morganii, Aeromonas hydrophila, Klebsiella pneumoniae, Proteus vulgaris, Pseudomonas fluorescens, Providencia rettgeri* and others [6–8]. Therefore, we decided to screen the intestinal microbiota of a Polish reptile: grass snake *Natrix natrix.* Grass snake (*Natrix natrix*) is a widespread species of reptile occupying most of Europe, as well as northern Africa and western Asia [20]. Grass snake occurs in a wide range of habitats, with a tendency to inhabit anthropogenic areas [21], thus it serves as a perfect model for epidemiological research of reptile-associated pathogens.

## Materials and Methods

### Sample Collection from Animals

Snakes used in the study were collected in the area of Kraków (S Poland; *N* = 30) and in Puszcza Niepołomicka *[Niepołomice Forest]* (S Poland; *N* = 15) in summer during the active season. Since microbiota of snakes is likely to be strongly affected by the absorptive state [22] and recent diet, we transferred the snakes to the laboratory to standardize sampling conditions. Snakes were kept solitarily in disinfected terraria, with water provided ad libitum, and fed once per week. Before sampling, all snakes were fed with captive rodents to equalize the diet composition and get insight into the composition of *Enterobacterales* representing the core microbiota of the snakes. In addition, before being offered to the snakes, rodents were kept in − 20 ℃ to deplete their microbiota. Each snake was sampled at least 1 week after feeding to ensure it is in the post-absorptive state [23]. Samples, in the form of cloacal swabs, were collected by placing a sterile swab inside the cloaca of a snake for 30 s with gentle rotational movements. Cloacal swabs were taken from 45 *N. natrix* individuals and then placed in the Amies medium at the Jagiellonian University in Kraków, Institute of Environmental Sciences by the Population Ecology Team based on the decision of the Regional Directorate for Environmental Protection in Kraków no. OP I.6401.21.2015.PKw, no. OP-I.6401.368.2016.PKw and decision of the Local Ethical Committee no. 132/2015 of 26 May, 2015 and 73/2017 of 16 February 2017. All applicable institutional and/or national guidelines for the care and use of animals were followed. All collected samples were delivered to the Department of Microbiology at the University of Wrocław.

### Bacteriological Examination

The collected swabs were incubated in LB Broth (Biocorp, Warsaw, Poland) at 37 °C for 24 h with shaking at about 200 rpm. Then, the cultures were plated onto MacConkey, *Salmonella*-*Shigella* and Nutrient Agar, (Biocorp, Warsaw, Poland) for 24 h at 37 °C. The isolated pure colonies were identified using MALDI-TOF MS in the Polish Collection of Microorganisms of the Hirszfeld’s Institute of Immunology and Experimental Therapy, Polish Academy of Sciences.

### MALDI-TOF MS Analysis

Pure single colonies of actively growing cultures were mixed to obtained a bacterial suspension in 300 μL of water, then fixed by the addition of 900 μL absolute ethanol, which was followed by extraction procedure as previously described [24, 25]. Bacterial strains were identified by MALDI-TOF MS and all analyses were performed with an UltrafleXtreme mass spectrometer (Bruker Daltonics, Germany) using the Biotyper 3.1 software and database containing 4613 entries. Identification by MALDI-TOF MS was repeated twice.

## Results

Nineteen species of Gram-negative bacteria were isolated from 45 animals (full list in Table 1). The most common species were: *Aeromonas hydrophila* (37.8%), *Morganella morganii* (26.7%), *Proteus vulgaris* (24.4%). *Salmonella* spp*.* were present in 10 cloacal swabs (22.2%). It is worth noting that among the bacteria isolated from reptiles the following species were less frequently identified: *Roultella ornithinolytica, R. planticola, Yokenella regensburgei, Leclercia adecarboxylata, Lelliottia amnigena.*

**Table 1:** Microbiota of forty-five *N. natrix* snakes

Bacterial species	Number of isolates and percentage (*N. natrix n* = 45)
*Aeromonas hydrophila*	17 (37.8%)
*Morganella morganii*	12 (26.7%)
*Proteus vulgaris*	11 (24.4%)
*Salmonella* sp.	10 (22.2%)
*Citrobacter braakii*	8 (17.8%)
*Providencia rettgeri*	7 (15.6%)
*Pseudomonas putida*	5 (11.1%)
*Aeromonas veronii*	4 (8.9%)
*Citrobacter freundii*	4 (8.9%)
*Klebsiella oxytoca*	3 (6.7%)
*Proteus hauseri*	3 (6.7%)
*Raoultella ornithinolytica*	3 (6.7%)
*Raoultella planticola*	2 (4.4%)
*Yokenella regensburgei*	2 (4.4%)
*Aeromonas ichthiosmia*	1 (2.2%)
*Alcaligenes faecalis*	1 (2.2%)
*Leclercia adecarboxylata*	1 (2.2%)
*Lelliottia amnigena*	1 (2.2%)
*Proteus penneri*	1 (2.2%)

All results from the MALDI-TOF analysis, including the scores are listed in Supplementary Table.

We compared the number of bacterial species per individual between two study sites, Kraków and Puszcza Niepołomicka, however, we did not find any significant differences (Mann–Whitney *U* test: *U* = 224.5; *Z* = 0.00; *P* = 1.0). The mean (2.13) and median (2) number of species per individual were the same at both study sites. The prevalence of *Salmonella* spp. in the two groups was comparable: 5 individuals out of 30 (17%) in Kraków; 5 individuals out of 15 (33%) in Puszcza Niepołomicka (Fischer exact test *P* = 0.1863).

## Discussion

To the best of our knowledge, so far there have only been two studies showing the composition of the cloacal microbiota of free-living snakes in Europe [7, 8]. Our study confirms the previous findings that free-living European snakes are carriers of Gram-negative bacteria, including human pathogens: *Salmonella* spp., *A. hydrophila, M. morganii, P. vulgaris* and other. However, there are many differences between the results obtained by our team and those published by Schmidt et al. and Lukać et al. [7, 8]. This indicates that more European studies are needed. Our results partly contradict those published by Schmidt et al. [7], as they indicated that the most common species of bacteria was *A. hydrophila*, which has been absent in the cloacal microbiota of *N. natrix* living freely in Germany. However, *A. hydrophila* has been present in *Vipera berus*, the second snake species studied by Schmidt et al. Also snakes used in Lukać et al. study [8] have been carriers of *A. hydrophila*. Other researchers (whose research did not concern free-living European snakes) have proven that *A. hydrophila* is often present in the microbiota of reptiles [26–29]. The second most common bacterial species that snakes did carry in our study was *M. morganii*. Here, we cannot find any correlation with the results of Schmidt et al., where the reptiles have been free of this pathogen. Also, Lukać et al. [8] has shown that free-living snakes in Croatia are free of *M. morganii.* However, again, other researchers have proven that *M. morganii* is present in the intestinal and oral flora of snakes [6]. The third most common species in cloacal microbiota of *N. natrix* in our study was *P. vulgaris,* and here we found correlation with Schmidt et al. studies, where 75.0% of examined *N. natrix* have been carriers of this bacterial species. In Lukac’s et al. studies *P. vulgaris* has also been present. In the case of *Salmonella* ssp.—the genus has been absent in the previously cited publication regarding German free-living *N. natrix*, however, 34.8% of free-living *V. berus* from the same study have been carriers of *Salmonella* spp. Croatian free-living snakes have been free of *Salmonella* isolates. On the other hand, Zając et al. has tested free-living Polish snakes (including *N. natrix*) for the presence of *Salmonella* spp. and 87.5% of them have been positive. In our studies, 22.2% of snakes were carriers of *Salmonella* spp.

One of the major differences between our study and the previously published records is the sampling regime. Schmidt et al. and Lukać et al. [7, 8] have collected samples immediately after snakes were captured. Therefore, the results could have been affected by the outside temperature prior to sampling and the absorptive state of snakes. In our case, snakes were kept in laboratory conditions standardized in terms of ambient temperature and were sampled in the post-absorptive state in each case. We recommend that such standardization prior to sampling should be considered as crucial in future studies, due to the known effect of temperature [30] and the absorptive state [22] on the microbiome. Particularly, the absorptive state seems to affect the microbiome in a very dynamic way [22], therefore a comparison of snakes that are kept in standardized conditions seems to be more reliable.

The most common bacterial species found by our team in the cloacal microbiota of *N. natrix* were human pathogens, however, infections caused by them are rare and mostly affect immunocompromised patients.

*Aeromonas hydrophila* is a very common bacteria species, widespread in water sources. The bacterium uses *quorum sensing* and forms biofilms, which are its most important virulence factors. *A. hydrophila* is an enteric human pathogen, at the same time being an opportunistic nosocomial pathogen, often resistant to antibiotics [31].

*Morganella morganii* is an opportunistic pathogen, a causative agent of urinary tract infection. The bacteria are very rarely human pathogens, being mostly isolated from immunocompromised patients [32].

*Proteus vulgaris* is widely distributed in the environment. It is an opportunistic human pathogen usually causing urinary tract infections [32, 33]. One of the most important virulence factors is its ability to form biofilm. Most of the infections having serious health consequences are connected with biofilm formation [34, 35].

*Salmonella* spp.—the 4th common bacterial species found as a component of Polish grass snake’s cloacal microbiota is an exclusively human pathogen. However, reptiles are asymptomatic carriers of these bacteria and they are not display any symptoms of salmonellosis. This phenomenon is still poorly explained, therefore, most of the microbiological studies focusing on reptile’s microbiota concern *Salmonella* spp.[1–5]. Among humans *Salmonella* ssp. is causative agent of salmonellosis, which is predominantly a gastrointestinal tract infection. Salmonellosis can also lead to severe infections, such as: bacteraemia, meningitis, bone infections, and urinary tract infections. Salmonellosis is a zoonosis and reptiles are among vectors for it. Most of the data regarding RAS are from the USA, where 6.0% of sporadic salmonellosis cases are caused by reptile and amphibian contact [5]. European studies are rare, however recently they have been more frequently undertaken [8, 9, 11, 12, 14, 35, 36]. Our studies confirm that grass snakes living freely in Poland are carriers of *Salmonella* spp., however the prevalence is much lower than in the case of exotic reptiles.

The prevalence of some bacterial species should be highlighted, even if it is a rare phenomenon. 15.6% of the tested snakes were carriers of *P. rettgeri.* This bacterium is a common species found in the environment (both water and land). However, recently there have been cases showing that *P. rettgeri* is also an opportunistic human pathogen, responsible for wound infections and even neonatal sepsis [37–39].

Considering the role of reptiles as carriers of human pathogens, it is worth pointing out that our study confirmed the presence of *R. ornithinolytica* and *R. planticola* as components of snake’s microbiota. *R. ornithinolytica* has so far been considered as a saprophytic bacterium, formerly known as *Klebsiella oxytoca*. However the application of MALDI-TOF MS allowed to differentiate between *Roultella* and *Klebsiella* [40]. In our study, we also used MALDI-TOF MS, which is a recommended technique for identification of bacteria, also of *Salmonella* spp.[41]. Owing to the use of MALDI-TOF MS for bacterial identification, it has been determined that *R. ornithinolytica* is an underreported, emerging hospital-acquired human pathogen responsible for osteomyelitis, meningitis, cerebral abscess, mediastinitis, pericarditis, conjunctivitis, and otitis [42].

*Yokenella regensburgei* is not well-known species, originally isolated from intestinal tracts of insects and reptiles [43]. As a relatively new species, often misidentified as *Salmonella* or *Hafnia, Y. regensburgei,* is not well-documented human pathogen. However, new techniques of identification, including MALDI-TOF MS, provide new information about this bacterium [44].

The bacteria present in *N. natrix* cloacal swabs seem to be the natural intestinal microbiota of the tested snakes. However, the species described above are human pathogens, so people having close contact with free-living *N. natrix* should be aware of the possible risk of transmission of these pathogens. The risk of infection is high for immunocompromised humans, small children (under 5 years old), elderly persons, and pregnant women and it can be enhanced by domestic animals as additional vectors having contact with free living snakes.

Unlike the publications of other researchers [7, 8, 11], our studies present the results of Gram-negative intestinal microbiota for the biggest group of free-living European snakes (*N* = 45). To the best of our knowledge, the results presented in this paper are the first on such a big population of free-living snakes in Europe.

## Electronic supplementary material

Below is the link to the electronic supplementary material.

Supplementary file1 (DOCX 20 kb)

## Data Availability

The datasets analyzed during the current study are available from Agnieszka Korzeniowska-Kowal (agnieszka.korzeniowska-kowal@hirszfeld.pl) on reasonable request.
