# Supplementary material for: Cloacal Gram-Negative Microbiota in Free-Living Grass Snake Natrix natrix from Poland
Source: Curr Microbiol. 2020 May 19;77(9):2166–71. doi: 10.1007/s00284-020-02021-3 (PMC7415037; doi:10.1007/s00284-020-02021-3)
Supplement: Supplementary file 1 — Supplementary file1 (DOCX 20 kb) [file 284_2020_2021_MOESM1_ESM.docx]

**Supplementary table.** Score values of MALDI-TOF analysis.

|  | **Analyte name** | **Organism (best match)** | **Score Value** |
| --- | --- | --- | --- |
| **1** | 2.1K | *Aeromonas veronii* | 2,418 |
| **2** | 2.2K | *Aeromonas hydrophila* | 2,508 |
| **3** | 8.1K | *Citrobacter braakii* | 2,286 |
| **4** | 8.2K | *Aeromonas hydrophila* | 2,301 |
| **5** | 8.3K | *Morganella morganii* | 2,435 |
| **6** | 10.1K | *Proteus vulgaris* | 2,278 |
| **7** | 10.2K | *Citrobacter braakii* | 2,345 |
| **8** | 12.1K | *Salmonella sp.* | 2,315 |
| **9** | 12.2K | *Aeromonas hydrophila* | 2,339 |
| **10** | 14.1K | *Lelliottia amnigena* | 2,019 |
| **11** | 14.2K | *Providencia rettgeri* | 2,528 |
| **12** | 14.3K | *Citrobacter freundii* | 2,379 |
| **13** | 16.1K | *Citrobacter braakii* | 2,317 |
| **14** | 16.2K | *Raoultella planticola* | 2,470 |
| **15** | 16.3K | *Providencia rettgeri* | 2,638 |
| **16** | 22.1K | *Aeromonas hydrophila* | 2,415 |
| **17** | 22.2K | *Morganella morganii* | 2,496 |
| **18** | 27.1K | *Aeromonas hydrophila* | 2,344 |
| 19 | 27.2K | *Proteus vulgaris* | 2,346 |
| **20** | 27.3K | *Morganella morganii* | 2,493 |
| **21** | 31.1K | *Providencia rettgeri* | 2,516 |
| **22** | 31.2K | *Citrobacter freundii* | 2,476 |
| **23** | 32.1K | *Morganella morganii* | 2,390 |
| **24** | 32.2K | *Citrobacter freundii* | 2,370 |
| **25** | 34K | *Aeromonas hydrophila* | 2,474 |
| **26** | 37K | *Proteus vulgaris* | 2,295 |
| **27** | 39.1K | *Salmonella sp.* | 2,314 |
| **28** | 39.2K | *Yokenella regensburgei* | 2,158 |
| 29 | 44.1K | *Morganella morganii* | 2,406 |
| **30** | 44.2K | *Aeromonas hydrophila* | 2,208 |
| 31 | 1.1S | *Providencia rettgeri* | 2,520 |
| 32 | 1.2S | *Salmonella sp.* | 2,258 |
| **33** | 1.3S | *Aeromonas hydrophila* | 2,499 |
| 34 | 4.1S | *Salmonella sp.* | 2,126 |
| 35 | 4.2S | *Citrobacter freundii* | 2,350 |
| 36 | 5S | *Aeromonas hydrophila* | 2,458 |
| 37 | 7.1S | *Salmonella sp.* | 2,214 |
| 38 | 7.2S | *Providencia rettgeri* | 2,658 |
| 39 | 11S | *Salmonella sp.* | 2,330 |
| 40 | 13.1S | *Aeromonas hydrophila* | 2,476 |
| 41 | 13.2S | *Morganella morganii* | 2,618 |
| 42 | 13.3S | *Salmonella sp.* | 2,266 |
| 43 | 13.4S | *Citrobacter braakii* | 2,345 |
| 44 | 15.1S | *Proteus hauseri* | 2,305 |
| 45 | 15.2S | *Citrobacter braakii* | 2,161 |
| 46 | 15.3S | *Morganella morganii* | 2,228 |
| 47 | 18.1S | *Yokenella regensburgei* | 2,278 |
| 48 | 18.2S | *Aeromonas hydrophila* | 2,457 |
| 49 | 18.3S | *Proteus penneri* | 2,258 |
| 50 | 20.1S | *Alcaligenes faecalis* | 2,112 |
| 51 | 20.3S | *Aeromonas hydrophila* | 2,386 |
| 52 | 25.1S | *Morganella morganii* | 2,527 |
| 53 | 25.2S | *Proteus vulgaris* | 2,468 |
| 54 | 28.1S | *Salmonella sp.* | 2,299 |
| 55 | 28.2S | *Citrobacter braakii* | 2,336 |
| 56 | 28.3S | *Morganella morganii* | 2,583 |
| 57 | 43.1S | *Morganella morganii* | 2,593 |
| 58 | 43.2S | *Aeromonas hydrophila* | 2,496 |
| 59 | 49.2S | *Raoultella ornithinolytica* | 2,402 |
| 60 | 49.3S | *Proteus vulgaris* | 2,399 |
| 61 | 50.1S | *Aeromonas ichthiosmia* | 2,293 |
| 62 | 50.2S | *Proteus vulgaris* | 2,364 |
| 63 | 50.3S | *Pseudomonas putida* | 2,263 |
| 64 | 52S | *Proteus vulgaris* | 2,188 |
| 65 | 3.1L | *Salmonella sp.* | 2,143 |
| 66 | 3.2L | *Proteus hauseri* | 2,361 |
| 67 | 3.3L | *Aeromonas hydrophila* | 2,407 |
| 68 | 3.4L | *Pseudomonas putida* | 2,024 |
| 69 | 6.1L | *Proteus hauseri* | 2,510 |
| 70 | 6.2L | *Klebsiella oxytoca* | 2,238 |
| 71 | 21.1L | *Pseudomonas putida* | 2,209 |
| 72 | 21.2L | *Raoultella planticola* | 2,271 |
| 73 | 24.1L | *Providencia rettgeri* | 2,530 |
| 74 | 24.2L | *Salmonella sp.* | 2,183 |
| 75 | 24.3L | *Citrobacter braakii* | 2,385 |
| 76 | 24.4L | *Proteus vulgaris* | 2,360 |
| 77 | 26L | *Aeromonas hydrophila* | 2,384 |
| 78 | 30.1L | *Citrobacter braakii* | 2,298 |
| 79 | 30.2L | *Aeromonas hydrophila* | 2,376 |
| 80 | 35.1L | *Providencia rettgeri* | 2,416 |
| 81 | 35.2L | *Proteus vulgaris* | 2,316 |
| 82 | 36L | *Proteus vulgaris* | 2,433 |
| 83 | 38.1L | *Morganella morganii* | 2,518 |
| 84 | 38.2L | *Aeromonas hydrophila* | 2,283 |
| 85 | 40.2L | *Pseudomonas putida* | 2,072 |
| 86 | 40.3L | *Raoultella ornithinolytica* | 2,378 |
| 87 | 40.4L | *Morganella morganii* | 2,475 |
| 88 | 45L | *Aeromonas veronii* | 2,328 |
| 89 | 46.1L | *Aeromonas veronii* | 2,212 |
| 90 | 46.2L | *Leclercia adecarboxylata* | 1,974 |
| 91 | 47.1L | *Pseudomonas putida* | 2,11 |
| 92 | 47.2L | *Klebsiella oxytoca* | 2,235 |
| 93 | 47.3L | *Raoultella ornithinolytica* | 2,182 |
| 94 | 47.4L | *Proteus vulgaris* | 2,357 |
| 95 | 48.1L | *Klebsiella oxytoca* | 2,328 |
| 96 | 48.3L | *Aeromonas veronii* | 2,374 |

**Meaning of Score Values**

| **Range 3,000-2,300**  **highly probable species identification** | **Range 2,299-2,000**  **secure genus identification, probable species identification** | **Range 1,999-1,700**  **probable genus identification** | **Range 1,699-0,000**  **not reliable identification** |
| --- | --- | --- | --- |
